# Assessing the combined effects of embryonic thermal conditioning and herbal feed additives on acquired thermotolerance of slow-growing poultry in a coastal climate

**DOI:** 10.3389/fphys.2026.1861569

**Published:** 2026-10-28

**Authors:** Nibedita Nayak, Subrat Kumar Bhanja, Eaknath B. Chakurkar, Amiya Ranjan Sahu, Santwana Palai, Ashok Kumar Pattanaik

**Affiliations:** ICAR-Central Coastal Agricultural Research Institute, Goa, India

**Keywords:** acquired thermotolerance, embryonic thermal conditioning, herbal feed additives, production performance, slow-growing poultry

## Abstract

This study aimed to enhance thermotolerance in CARI-Debendra chickens under hot and humid climatic conditions by integrating embryonic thermal conditioning (ETC) and early post-hatch supplementation with natural herbal feed additives. About 224 chicks from the control and ETC groups were further divided into herbal-supplemented and nonsupplemented groups for 12 weeks using a 2 × 2 factorial design. Turmeric (*Curcuma longa*) and ginger (*Zingiber officinale*), screened using Fourier-transform infrared spectroscopy (FTIR) and gas chromatography–mass spectrometry (GC-MS), were added to the diet at a 1% inclusion level each. ETC group birds had higher body weight (*p* < 0.01) but lower thigh % (*p* < 0.05), spleen weight (*p* < 0.05), and intestinal weight (*p* < 0.01) than control birds. An interaction effect between ETC and posthatch herbal supplementation on body weight was observed only at the third and fifth weeks. The phytochemical profiling of turmeric (*Curcuma longa*) confirmed the presence of curcumin, 1-decanol, 2-hexyl- and 1-heptacosanol whereas ginger (*Zingiber officinale*) showed the presence of gingerols and shogaols, zingiberene, limonene, ethanone, 1-(2,2-dimethylcyclopentyl)-,1-but-2-enylpyrrolidine, *N*-butyric acid 2-ethylhexyl ester, which exhibit antioxidant and immunomodulatory activities and may serve as potential alternate candidates for further investigation to improve growth performance, egg quality, and economic efficiency in poultry. ETC had effects only on thigh weight, spleen weight, and intestinal weight without altering intestinal architecture compared with the control. Serum biochemical indicators, including thyroxine (T4) levels and creatine kinase activity, were elevated in the ETC + herbal supplementation group, while the heterophil-to-lymphocyte (H/L) ratio and triiodothyronine (T3) levels were lower, suggesting improved thermotolerance. In conclusion, the combination of ETC (39.5 °C and 60%–70% relative humidity for 5 h daily during embryonic days 11 to 16) and natural feed additives like turmeric and ginger improved heat resilience, immune competence, and productivity of CARI-Debendra poultry under tropical conditions.

## 1 Introduction

High ambient temperatures, particularly when combined with elevated humidity, pose serious challenges to poultry health, productivity, and welfare (Vandana and Sejian, 2018). These effects are amplified in coastal regions, where high relative humidity impairs evaporative cooling, further increasing heat load on birds (Reay, 2019). The Temperature-humidity index (THI) effectively reflects heat stress severity, with Nayak et al. (2023) reporting that rising THI levels significantly increase thermal stress in slow-growing poultry varieties.

Poultry are susceptible to heat stress owing to their feather coverage, lack of sweat glands, and high basal body temperature. Backyard systems, particularly those involving slow-growing improved varieties like CARI-Debendra, expose birds to prolonged environmental stress. This compromises productivity, reproduction, immune response, gut health, and gene expression, while raising the cost of mitigation (Rajkumar et al., 2015). With global temperatures projected to rise by over 1.5 °C by the early 2030s (IPCC, 2021), effective and sustainable adaptation and acclimatisation strategies are urgently needed. Singh et al. (2023) reviewed the growth rate of indigenous birds in coastal states of India and stressed the adverse effects of genetic and environmental factors on performance.

A holistic approach combining genetic selection, nutritional support, and environmental management is recommended for heat stress mitigation (Biswal et al., 2022). Haunshi et al. (2025) also studied the difference between dwarf and normal Nicobari fowl and found that dwarfism was associated with decreased eighth-week body weight, shank length, and egg production. Among nutritional interventions, phytogenic feed supplementation and embryonic thermal conditioning have shown promise as strategies to improve thermotolerance. Embryonic thermal conditioning (ETC) is a prehatch strategy involving controlled thermal exposure during embryogenesis, typically between embryonic days 7 and 16, at 39.5 °C and 65% RH for 12 h/day. This induces long-term thermotolerance by adjusting the thermoregulatory set-point and reducing metabolic heat production posthatch, without affecting hatchability (Yahav et al., 2004; Loyau et al., 2014). Late-stage conditioning (E16–E18) yields only short-term benefits (Collin et al., 2007), while intermittent ETC positively influences muscle development and performance (Piestun et al., 2009). Thermal conditioning during embryonic and early hatch stages provides greater adaptability to heat stress in adult stages through TC-mediated epigenetic programming, which also protects against multiple stressors (Sajjanar et al., 2025).

Phytobiotics—plant-derived additives from herbs, spices, and extracts—offer antimicrobial, antioxidant, immunomodulatory, and gut health benefits without the risks associated with antibiotics (Kikusato, 2021). Commonly used herbs include turmeric, ginger, moringa, tulsi, and kalmegh. Turmeric is rich in curcumin and helps stabilise gut microbiota and improve growth performance in broilers and slow-growing chickens (Govindarajan and Stahl, 1980; Lorenzi and Matos, 2002; Wang et al., 2015). Ginger, containing gingerol, has demonstrated antioxidant, hypocholesterolemic, and immunomodulatory effects, with studies reporting improved egg quality and bird health at 0.5–0.75% inclusion levels (Akbarian et al., 2011; Dieumou et al., 2009; Zhao et al., 2011; Abd El-Hack et al., 2020). The use of natural feed additives has received attention due to their bioactive compounds that have beneficial effects on growth, health status, and thermoregulatory properties (Kujero et al., 2024).

This study investigates the combined effect of ETC and dietary supplementation of local cultivars of turmeric and ginger (1% each) on the growth performance and thermotolerance of slow-growing poultry under coastal tropical conditions. The outcomes aim to offer an integrative model for sustainable poultry production through epigenetic, nutritional, and environmental interventions.

## 2 Material and methods

### 2.1 Experimental site and meteorological observations

The experiment was conducted at the institute poultry farm of the Indian Council of Agricultural Research-Central Coastal Agricultural Research Institute (ICAR-CCARI), Goa, at geographical coordinates of 15.2993°N and 74.1240°E. This coastal ecosystem is characterised by a hot and humid climate with an average annual rainfall of 3,405 mm for over 20 years. The monthly mean bright sunshine hours during the study period ranged between 7.6 and 9.0 h. The monthly average maximum and minimum temperatures during the study period ranged from 35.7 °C to 34.4 °C and 20.5 °C to 24.1°C, respectively. Relative humidity (RH %) varied from 57.6 ± 11 to 70.4 ± 5.3 during the period, resulting in a THI ranging from 76.2 ± 1.7 to 81.3 ± 1.4, indicating moderate discomfort. THI was calculated using the following formula: THI = 0.8 Tdb + RH (Tdb − 14.3)/100 + 46.3, where Tdb is dry-bulb temperature (Moraes et al., 2008).

### 2.2 Embryonic thermal conditioning and treatment groups

Fertile CARI-Debendra eggs were incubated using two protocols: (i) Control—incubation at 37.5 °C, 60% RH (setter), and 37 °C, 75% RH (hatcher); and (ii) ETC—incubation at 39.5 °C and 60%–70% RH for 5 h/day from embryonic days 11–16. After candling, 300 fertile eggs were selected and equally distributed (*n* = 150 per treatment, control and ETC). Posthatch, chicks from each treatment group were randomly allotted to two subgroups (no herbal supplementation and herbal supplementation), thus forming four treatment groups: Control, Control + Herbal, ETC, and ETC + Herbal, each comprising four replicates of 14 chicks.

### 2.3 Experimental birds and design

CARI-Debendra, a slow-growing dual-purpose poultry breed developed for hot and dry climates, was used for this study. The experiment continued for a 12-week period, including 4 weeks of brooding, as outlined in **Table 1**. The experimental design for this study was a 2 × 2 factorial design, with each experimental unit randomly assigned to a treatment. The prevailing temperature and humidity data were recorded hourly using a Lascor EL-USB2-LCD+ sensor to monitor the THI for each treatment group.

**Table 1:** Experimental design for pre- and posthatch herbal supplementation.

Treatments	Prehatch thermal conditioning	
	No thermal conditioning (control)	Embryonic thermal conditioning (ETC)
Posthatch herbal supplementation		
NPHHS	T1	T3
EPHHS (supplementation of a combination of turmeric and ginger at a1% level in feed)	T2	T4

*ETC*, embryonic thermal conditioning; *NPHHS*, no posthatch herbal supplementation; *EPHHC*, early posthatch herbal supplementation; *T1*, control + NPHHS; *T2*, control + EPHHS; *T3*, ETC + NPHHS; *T4*, ETC + EPHHS.

### 2.4 Preparation of phytogenic feed additive blend

Rhizomes of turmeric (Prathibha) and ginger (Mahima), sourced from Goa, India, were cleaned, boiled, shade-dried, oven-dried at 37 °C, milled, and sieved (3 mm). The fine powder was used for feed supplementation. Proximate analysis of turmeric and ginger using the AOAC (2005) method revealed moisture (11.07%, 0.39%), crude protein (0.6%, 0.64%), crude fibre (5.11%, 20.9%), crude fat (3.16%, 1.78%), Ca (2.24%, 2.78%), and P (1.30%, 1.78%) on a dry basis, respectively. Herbal groups received a 1% turmeric–ginger blend in addition to the balanced phase diet.

### 2.5 Quantitative and qualitative screening of phytochemicals

UV-Vis spectrophotometry (Perkin Elmer Lambda 365), scanning over the range of 1,100–190 nm to determine the λ-max, and Fourier transform infrared (FTIR) analysis (Perkin Elmer UTAR TWO), scanning over the range of 4,000–450 cm^−1^ to identify potential functional groups, were performed on the rhizomes of turmeric and ginger. Active compound quantification was carried out using gas chromatography-mass spectrometry (GC-MS; PerkinElmer Clarus 590), which provided retention times, peak areas, and percentages of phytocompounds.

### 2.6 Basal diet and feeding

Birds received a corn-soya basal diet formulated according to the Indian Council of Agricultural Research (ICAR 2013): phase I (0–6 weeks): 2,800 Kcal/kg ME, 21% CP; phase II (7–12 weeks): 2,700 Kcal/kg ME, 18% CP. The feed formulations and nutrient composition are provided in **Table 2**. Weekly feed intake and growth were recorded. The lighting regimen consisted of 23 h of continuous light during the first phase (0–6 weeks) and natural light during the second phase (7–12 weeks). The birds were reared under optimal welfare conditions with *ad libitum* access to clean water throughout the trial.

**Table 2:** Ingredient composition and chemical analysis of feed for improved indigenous CARI-Debendra birds.

Ingredients (%)	Phase 1	Phase 2
Maize	53	44.20
Soybean	28.5	8.00
Deoiled rice bran	11	24
Rice polish		3.5
Meat cum bone meal	5	4.5
Rape seed meal		5
Sunflower seed meal		7.5
Limestone	0.54	1.00
Di-calcium phosphate	0.75	0.8
Salt	0.3	0.3
Lysine	0.30	0.3
Methionine	0.11	0.10
Vit premix	0.5	0.8
Total	100	100
Crude protein	21	18.47
Metabolizable energy (kcal/kg)	2,818	2,700
Calcium	1.02	1.08
Avail. phosphorus	0.59	0.56
l-Lysine	1.52	0.91
dl-Methionine	0.54	0.37

Herbal feed additives (turmeric and ginger) were incorporated into the feeding regimen during this phase at a 1% inclusion level each.

### 2.7 Thermoregulatory assessment

Thermoregulatory indices measured included respiration rate, rectal temperature, and body surface temperature. Measurements were taken at 11:00 AM and 4:00 PM on the day from five birds per replicate. Rectal temperature was measured using a clinical thermometer inserted up to 1 cm deep in the rectum after gently handling the chicks individually. A thermal scanner was used for assessing external body temperature at the feet, shank, back, neck, and comb (Souza-Junior et al., 2019). Respiratory rate (panting breaths over 15 s × 4) was counted at both time points.

### 2.8 Performance traits

Weekly body weight of individual birds was measured over 12 weeks using a digital balance with 0.1 g accuracy. Feed conversion ratio at different weekly intervals was calculated by dividing the total feed consumed by weight gain in kilogrammes. Daily monitoring of the birds was carried out, and mortality, if any, was recorded. Extra chicks were maintained group-wise under their specific environmental conditions, and mortality, if any, was compensated for with extra chicks from the respective groups.

### 2.9 Carcass characteristics

After the 12th week, eight birds of each sex (one male and one female from each replicate) per treatment were selected with body weights close to the mean for carcass studies. Selected birds were kept off-feed overnight before slaughter; however, during that period, drinking water was offered to them. Carcass characteristics such as carcass yield, cut-up parts, giblets, and intestinal weight were recorded and expressed as a percentage of live weight.

### 2.10 Haematological parameters

At 6 weeks, 2 mL of blood was collected from the wing vein of eight birds per treatment. Haemoglobin was estimated spectrophotometrically. RBC counts were determined using a hemacytometer. Heterophil-to-lymphocyte (H/L) ratios were estimated using Giemsa-stained blood smears (Lucas and Jamroz, 1961) and differential leukocyte counts (approximately 100 cells) on each slide at × 100 magnification.

### 2.11 Immune organ metrics and immune response

At 6 and 12 weeks, eight birds per treatment were selected randomly for slaughter. Spleen, bursa, and thymus weights were recorded and expressed as a percentage of body weight. Cell-mediated immunity was assessed using the cutaneous basophil hypersensitivity (CBH) response to phytohemagglutinin type-P (PHA-P) at 6 weeks (Corrier and DeLoach, 1990).

### 2.12 Gut biometry and histomorphometry of small intestine

The birds slaughtered for immune organ study at 6 and 12 weeks of age were used to study the intestinal biometrics. Intestinal contents were flushed with PBS, and total lengths of the intestine and segments (duodenum, jejunum, ileum, caeca) were measured. Tissue samples were fixed in 10% formalin, sectioned (5 µm), stained (H&E), and analyzed for villus height, crypt depth, villus:crypt ratio, and surface area using MagVision 4.0 software (Uni et al., 1999).

### 2.13 Biochemical parameters

Blood was collected from eight birds per treatment group in the sixth week. The samples were allowed to stand for two hours before centrifugation at 3,000 rpm for 10 min at 4°C to separate serum for biochemical analysis. Different biochemical parameters, including the activities of enzymes creatine kinase (CK), lactate dehydrogenase (LDH), and the levels of triiodothyronine (T3), thyroxine (T4), and thyroid-stimulating hormone [TSH], were measured using the respective assay kits (Coral Clinical Systems Pvt. Ltd., Verna Industrial Estate, Goa). T3 and T4 were estimated according to the instructions provided with the respective kits (Mindray, Andheri East, Mumbai, Maharashtra).

### 2.14 Statistical analysis

Data were analyzed using SPSS statistical software (V.20.0), and differences in the sample means were compared using Duncan’s multiple range test (Duncan, 1955). Two-way analysis of variance (ANOVA) was used to study the interaction between main effects. The primary factor for this study was prehatch thermal conditioning, whereas the posthatch thermal conditioning served as a secondary factor. Feed conversion ratio (FCR) and biochemical parameters—including enzyme activities and hormone concentrations—were assessed using one-way ANOVA with a significance level set at *p* < 0.05.

## 3 Results

### 3.1 Quantitative and qualitative screening of phytochemicals through UV-Vis, FTIR, and GC-MS

The phytochemical screening of local cultivars (region-specific) of turmeric and ginger was carried out using UV-Vis and FTIR spectroscopy and GC-MS analysis. The UV-Vis spectra of turmeric showed absorbance between 300 and 500 nm, confirming the presence of curcumin, while ginger exhibited peaks at 230–280 nm, indicating the presence of gingerols and shogaols.

The FTIR analysis of turmeric revealed characteristic peaks at 3,360.33, 3,281.3, 2,919.34, 1,629.35, 1,603.27, and 1,074.99 cm^−1^ corresponding to curcumin. Ginger showed prominent peaks at 3,281.27 cm^−1^ (hydroxyl/phenolic groups), 2,924.99 cm^−1^ (methylene C–H), and 2,115.76–2,198.98 cm^−1^ (aromatic compounds). Peaks at 1,638.85 cm^−1^ were associated with terpenes such as zingiberene and limonene. The spectral data and compound mapping are presented in **Figures 1**, **2**. The bioactive compounds in turmeric and ginger analysed through GC-MS are presented in **Tables 3**, **4**.

**Figure 1:**
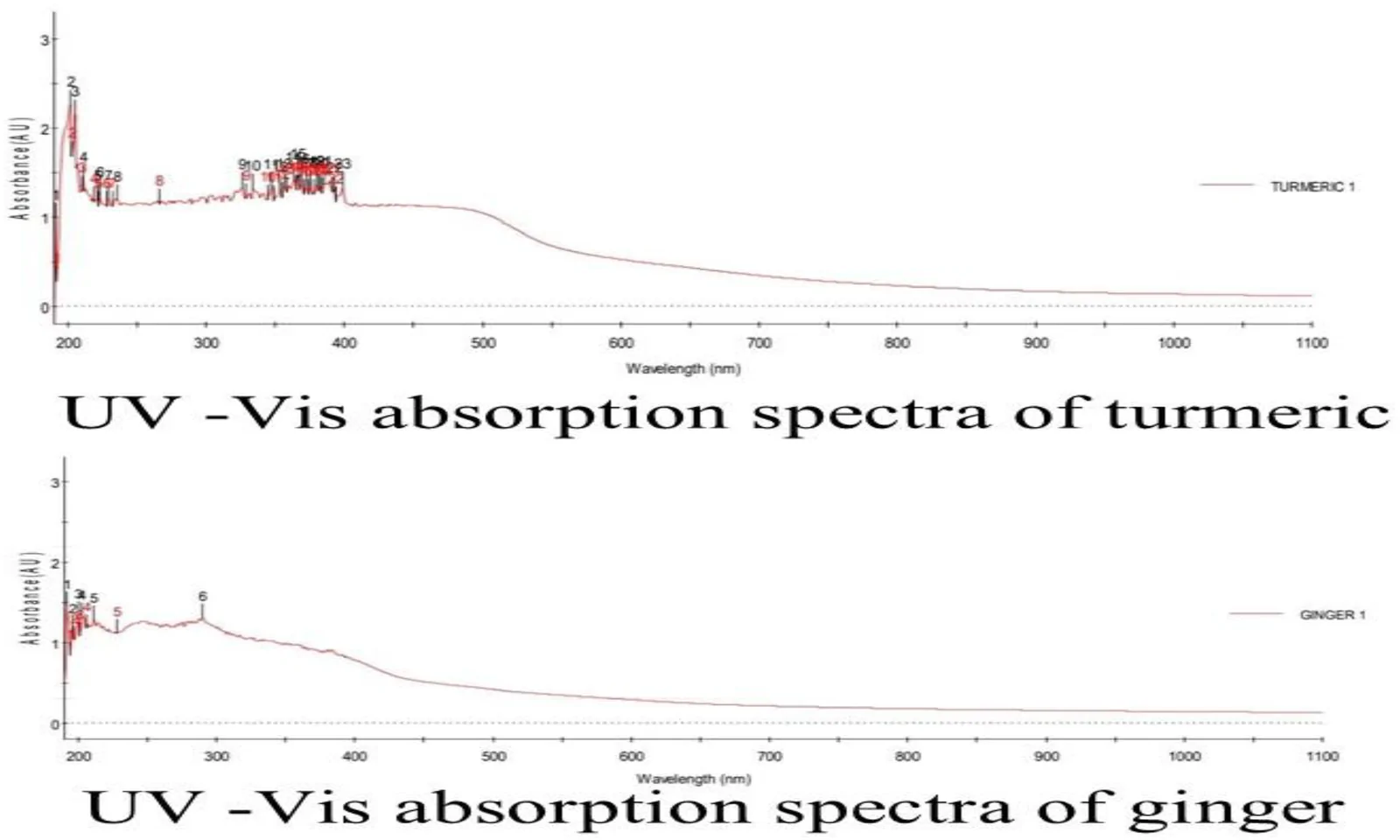
UV-Vis absorption spectra of turmeric and ginger rhizomes.

**Figure 2:**
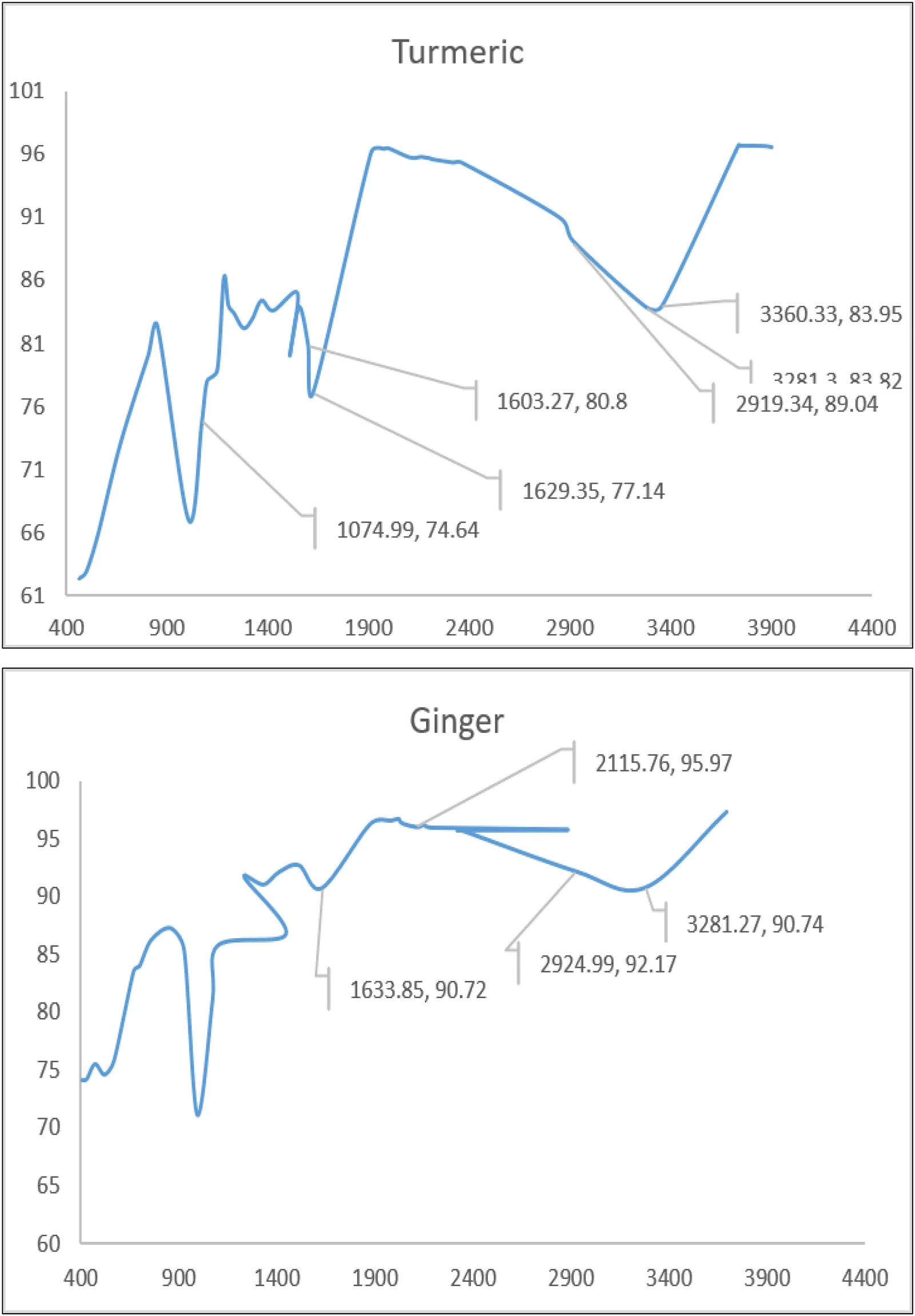
FTIR spectrum of turmeric and ginger rhizomes.

**Table 3:** GC-MS quantification of active compounds of *Curcuma longa* (turmeric).

Sl. no.	Active compound names	M.W.
1	17-Pentatriacontene	490
2	Acetic acid, 3,7,11,15-tetramethylhexadecyl ester	340
3	3,7,11,15-Trimethylhexadecanoic acid, 2,2,2-trifluoroethyl ester	394
4	1-Decanol, 2-hexyl-	242
5	Oxirane, [(dodecyloxy)methyl]-	242
6	1-Decanol, 2-hexyl-	242
7	1-Dodecanol, 2-octyl-	298
8	Cyclotetradecane, 1,7,11-trimethyl-4-(1-methylethyl)-	280
9	Nonacos-1-ene	406
10	1-Heptacosanol	396

**Table 4:** GC-MS quantification of active compounds in *Zingiber officinale* (ginger).

Sl. no.	Compound name	M.W.
1	Sulfurous acid, bis(2-pentyl) ester	222
2	Ethanone, 1-(2,2-dimethylcyclopentyl)-	140
3	1-But-2-enylpyrrolidine	125
4	3,5-Dimethyl-2-octanol	158
5	*N*-butyric acid 2-ethylhexyl ester	200
6	Heptan-2-OL, 5-(2-tetrahydrofurfuryl)-	200
7	Carbonic acid, neopentyl 2-ethylhexyl ester	244
8	1,2-Cyclohexanediol, 1-methyl-, trans-	130
9	2-Ethyl-5-propylcyclopentanone	154
10	Nonane, 4-methyl-5-propyl	184
11	*N*-butyric acid 2-ethylhexyl ester	200
12	Decyl pentyl ether	228
13	Fumaric acid, di(2-methylallyl) ester	224
14	Carbonic acid, 2-ethylhexyl octyl ester	286
15	Formamide, *n*-(4,6-diamino-5-pyrimidinyl)-	153
16	3-Nonen-2-one, 3-ethyl	168
17	Carbonic acid, 2-ethylhexyl nonyl ester	300
18	Carbonic acid, octyl vinyl ester	200
19	3-Cyclopentylpropionic acid, 2-ethylhexyl ester	254

### 3.2 Hatching parameters

ETC had no adverse impact on fertility (77.05% vs. 72.23%) or hatchability (63.53% vs. 63.23%) in CARI-Debendra birds. However, ETC significantly (*p* ≤ 0.05) improved hatch weight (35.05 g ± 0.34 g vs. 33.31 g ± 0.24 g) and advanced hatching by 10 h (492 h vs. 502 h in controls).

### 3.3 Thermotolerance parameters

At hatch only, control birds showed significantly (*p* ≤ 0.05) lower rectal (104.53°F ± 0.17°F vs. 105.62°F ± 0.10°F) and surface temperatures (93.90°F ± 0.26°F vs.98.37°F ± 0.27°F) compared with ETC. These differences in rectal temperatures and surface temperatures diminished with age and were within normal values as specified for chickens. Respiration rates were observed in the range of 46.10–51.46 breaths/min and remained statistically similar across treatments.

### 3.4 Performance traits

ETC birds exhibited significantly improved (*p* ≤ 0.05) body weights from hatch through 12 weeks (**Table 5**). Birds supplemented with turmeric and ginger (1% each) also showed enhanced growth, particularly at 2, 3, 5, and 6 weeks. FCR was unaffected by treatment interactions (**Table 6**). However, ETC led to a significantly lower (*p* ≤ 0.05) thigh weight, while other carcass traits and dressing percentage remained unaffected (**Table 7**).

**Table 5:** Effect of different technological interventions during embryonic and posthatch periods on weekly body weight of CARI-Debendra chicken (*n* = 56).

Effects	Treatments	Body weight											
		First week	Second week	Third week	Fourth week	Fifth week	Sixth week	Seventh week	Eighth week	Ninth week	10th week	11th week	12th week
Main effects													
Prehatch manipulation	Control	76.0^a^	127.4^a^	190.8^a^	291.4^a^	420.9^a^	573.0^a^	731.5^a^	883.7^a^	1,016.5^a^	1,168.8^a^	1,306.3^a^	1,478.6^a^
	ETC	80.3^b^	143.4^b^	232.3^b^	351.9^b^	510.9^b^	675.3^b^	841.0^b^	1,027.8^b^	1,171.7^b^	1,327.9^b^	1,452.7^b^	1,586.0^b^
	*p*-value	0.037^*^	0.01^**^	0.01^**^	0.01^**^	0.01^**^	0.01^**^	0.01^**^	0.01^**^	0.01^**^	0.01^**^	0.01^**^	0.01^*^
Posthatch manipulation	NPHHS	76.7	128.5^a^	198.5^a^	307.7	448.0^a^	599.9^a^	768.4	929.2	1,071.3	1,216.7	1,344.3	1,498.6
	EPHHS	78.8	138.0^b^	214.3^b^	327.3	480.1^b^	644.1^b^	797.7	959.1	1,093.0	1,249.7	1,371.1	1,542.1
	*p*-value	0.465	0.018^*^	0.016^*^	0.105	0.027^*^	0.024^*^	0.052^*^	0.328	0.478	0.488	0.564	0.562
**Interaction effects**	Control + NPHHS	75.5	126.3^a^	189.5	290.5	421.8	566.6	729.1	882.2	1,019.1	1,159.8	1,298.4	1,466.6
	Control + EPHHS	76.3	124.9^a^	188.2	288.7	424.5	586.1	742.7	892.1	1,036.2	1,193.8	1,325.2	1,514.7
	ETC + NPHHS	78.6	132.2^a^	213.2	335.7	494.8	656.3	849.4	1,029.1	1,179.1	1,338.0	1,457.3	1,583.1
	ETC + EPHHS	82.3	155.6^b^	249.4	379.1	546.5	715.5	872.5	1,054.0	1,182.1	1,337.6	1,450.9	1,598.7
	*p*-value	0.954	0.056^*^	0.016^*^	0.103	0.038^*^	0.316	0.604	0.895	0.914	0.886	0.964	0.848
	SEM	0.55	1.42	2.49	3.77	5.70	7.12	7.44	9.50	11.33	12.66	14.12	17.89

Means with the same superscript in a column do not differ significantly.

*ETC*, embryonic thermal conditioning; *NPHHS*, no posthatch herbal supplementation; *EPHHS*, early posthatch herbal supplementation; *n*, number of observations in each group.

^*^ *p* < 0.05; ^**^p < 0.01—levels of significance.

Interaction effect is the interaction between pre-hatch thermal conditioning and post-hatch performances.

**Table 6:** Effect of different technological interventions during the posthatch period on feed conversion ratio of CARI-Debendra chicken (*n* = 4).

Treatments	Period-wise FCR			
	0–4 weeks	4–8 weeks	8–12 weeks	1–12 weeks
NPHHS	2.21 ± 0.03	2.48 ± 0.05	4.00 ± 0.10	3.02 ± 0.11
Control + EPHHS	1.99 ± 0.04	2.19 ± 0.04	4.02 ± 0.14	2.63 ± 0.05
ETC + NPHHS	2.05 ± 0.05	2.55 ± 0.05	4.16 ± 0.07	2.91 ± 0.10
ETC + EPHHS	2.00 ± 0.03	2.56 ± 0.10	4.21 ± 0.08	2.88 ± 0.06

*n*, number of observations in each group; *ETC*, embryonic thermal conditioning; *NPHHS*, no posthatch herbal supplementation; *EPHHS*, early posthatch herbal supplementation.

**Table 7:** Effect of different technological interventions during the embryonic and posthatch periods on carcass characteristics of CARI-Debendra chicken (*n* = 8).

Effects	Treatments	Carcass characteristics													
		LW (g)		NW%	DW%	EW%	Breast%	Back%	Thigh%	Drumstick %	Wing%	Neck%	Liver%	Gizzard%	Heart%
Main effects															
Prehatch manipulation	Control		1,459.16	95.62	71.7	53.67	25.23	21.67	18.24^a^	16.76	11.04	5.56	3.97	5.21	0.91
	ETC		1,608.16	92.44	74.90	53.80	26.52	19.37	16.83^b^	17.38	10.46	5.74	3.61	4.72	0.80
	*p*-value		0.15	0.08	0.10	0.94	0.36	0.06	0.05^*^	0.70	0.54	0.53	0.31	0.36	0.21
Posthatch manipulation	NPHHS		1,540.00	91.93	73.54	52.31	24.58	21.25	17.72	16.22	11.69	5.45	3.63	4.82	0.84
	EPHHS		1,552.87	96.03	73.47	54.52	25.55	20.45	16.99	17.33	9.94	5.71	3.33	4.39	0.92
	*p*-value		0.93	0.19	0.95	0.50	0.25	0.62	0.53	0.75	0.31	0.59	0.12	0.16	0.50
**Interaction effects**	Control + NPHHS		1,466.25	95.24	71.94	53.02	23.07	23.40	18.30	17.45	11.71	5.30	3.69	4.87	0.86
	Control + EPHHS		1,485.00	95.39	72.10	53.83	23.74	20.83	18.45	14.82	9.90	5.65	3.55	4.69	1.01
	ETC + NPHHS		1,613.75	88.63	75.13	51.60	26.10	19.09	17.14	14.99	11.68	5.61	3.57	4.77	0.83
	ETC + EPHHS		1,620.75	92.86	74.83	55.21	27.37	20.07	15.54	19.83	9.98	5.77	3.11	4.10	0.83
	*p*-value		0.99	0.36	0.97	0.79	0.15	0.44	0.27	0.17	0.66	0.94	0.88	0.86	0.74
	SEM		47.17	0.95	0.88	0.78	0.74	0.78	0.36	0.80	0.44	0.12	0.18	0.26	0.03

Means with the same superscript in a column do not differ significantly.

*ETC*, embryonic thermal conditioning; *NPHHS*, no posthatch herbal supplementation; *EPHHS*, early posthatch herbal supplementation; *n*, number of observations in each group; *LW*, live weight; *NW*, New York weight; *DW*, dressed weight; *EW*, eviscerated weight.

^*^ *p* < 0.05—level of significance.

Interaction effect is the interaction between pre-hatch thermal conditioning and post-hatch performances.

### 3.5 Haematological parameters

Herbal-supplemented groups had lower heterophil but higher lymphocyte counts, resulting in moderate H/L ratios compared to the ETC control. WBC counts were numerically higher in the ETC-herbal group (**Table 8**), indicating enhanced immune activation.

**Table 8:** Effect of different technological interventions during the embryonic and posthatch periods on haematological parameters of CARI-Debendra chicken (*n* = 8).

Effects	Treatments	Haematological parameters						
		Hb (g/dl)	WBC (10^4^/L)	RBC (10^12^/L)	Heterophil%	Eosinophil%	Lymphocyte%	H/L
Main effects								
Prehatch manipulation	Control	13.97	68,378.57	2.82	40.95	3.00	56.04	0.85
	ETC	14.46	72,024.58	2.60	42.66	1.66	55.66	0.93
	*p*-value	0.43	0.09	0.26	0.79	0.16	0.99	0.58
Posthatch manipulation	NPHHS	14.42	68,692.30	2.94	49.46	1.61	48.92	1.08
	EPHHS	13.91	70,933.75	2.37	44.62	3.37	52.00	1.07
	*p*-value	0.69	0.54	0.06	0.07	0.16	0.09	0.69
**Interaction effects**	Control + NPHHS	14.44	65,780.00	2.88	45.40	1.80	52.80	0.88
	Control + EPHHS	14.05	68,068.75	2.44	43.00	4.50	52.50	1.05
	ETC + NPHHS	14.41	70,512.50	2.97	52.00	1.50	46.50	1.21
	ETC + EPHHS	13.78	73,798.75	2.31	46.25	2.25	51.50	1.11
	*p*-value	0.31	0.71	0.31	0.45	0.63	0.47	0.45
	SEM	0.26	1,132.43	2.70	2.31	0.42	2.40	0.08

*n*, number of observations in each group; *ETC*, embryonic thermal conditioning; *NPHHS*, no posthatch herbal supplementation; *EPHHS*, early posthatch herbal supplementation.

Interaction effect is the interaction between pre-hatch thermal conditioning and post-hatch performances.

### 3.6 Immune organ metrics and immune response

Neither embryonic nor post-hatch herbal supplementation significantly influenced relative weights of immune organs, particularly those of the thymus and bursa, at 6 and 12 weeks, or cutaneous hypersensitivity responses at 6 weeks (**Table 9**). However, a reduced spleen weight was observed in the ETC group at 6 weeks, indicating potential modulation of immune organ development.

**Table 9:** Effect of different technological interventions during the embryonic and posthatch periods on immune response of CARI-Debendra chicken (*n* = 12).

Effects	Treatments	Sixth week			12th week			Foot index (mm)
		Spleen	Bursa	Thymus	Spleen	Bursa	Thymus	
Main effects								
Prehatch manipulation	Control	0.18^a^	0.45	0.71	0.21	0.20	0.61	0.23
	ETC	0.17^b^	0.47	0.51	0.19	0.15	0.53	0.39
	*p*-value	0.019^*^	0.323	0.668	0.194	0.138	0.150	0.09
Posthatch manipulation	NPHHS	0.17	0.47	0.53	0.19	0.19	0.62	0.31
	EPHHS	0.18	0.51	0.65	0.19	0.15	0.53	0.39
	*p*-value	0.840	0.258	0.970	0.276	0.590	0.680	0.50
**Interaction effects**	Control	0.18	0.48	0.67	0.20	0.26	0.69	0.21
	Control + NPHHS	0.16	0.48	0.70	0.20	0.13	0.56	0.36
	ETC + NPHHS	0.17	0.46	0.40	0.17	0.14	0.55	0.39
	ETC+ EPHHS	0.18	0.54	0.60	0.17	0.16	0.50	0.41
	*p*-value	0.30	0.39	0.44	0.24	1.09	0.17	0.70
	SEM	0.01	0.02	0.04	0.01	0.02	0.03	0.05

Means with the same superscript in a column do not differ significantly.

*ETC*, embryonic thermal conditioning; *NPHHS*, no posthatch herbal supplementation; *EPHHS*, early posthatch herbal supplementation; *n*, number of observations in each group.

^*^ *p* < 0.05—significance level.

Interaction effect is the interaction between pre-hatch thermal conditioning and post-hatch performances.

### 3.7 Gut biometry and histomorphometry of small intestine

The microarchitecture of small intestinal segments, including villus length, villus width, crypt depth, and villous surface area in the ETM and control groups, is presented in **Figures 3**, **4**. Except for lower (*p* < 0.05) intestinal weight in the ETC group, no significant differences were observed in gut length or the sizes of the duodenum, jejunum, ileum, and cecum across treatments (**Table 10**). Similarly, villus height and crypt depth at 6 and 12 weeks remained consistent among all groups, indicating comparable intestinal development.

**Figure 3:**
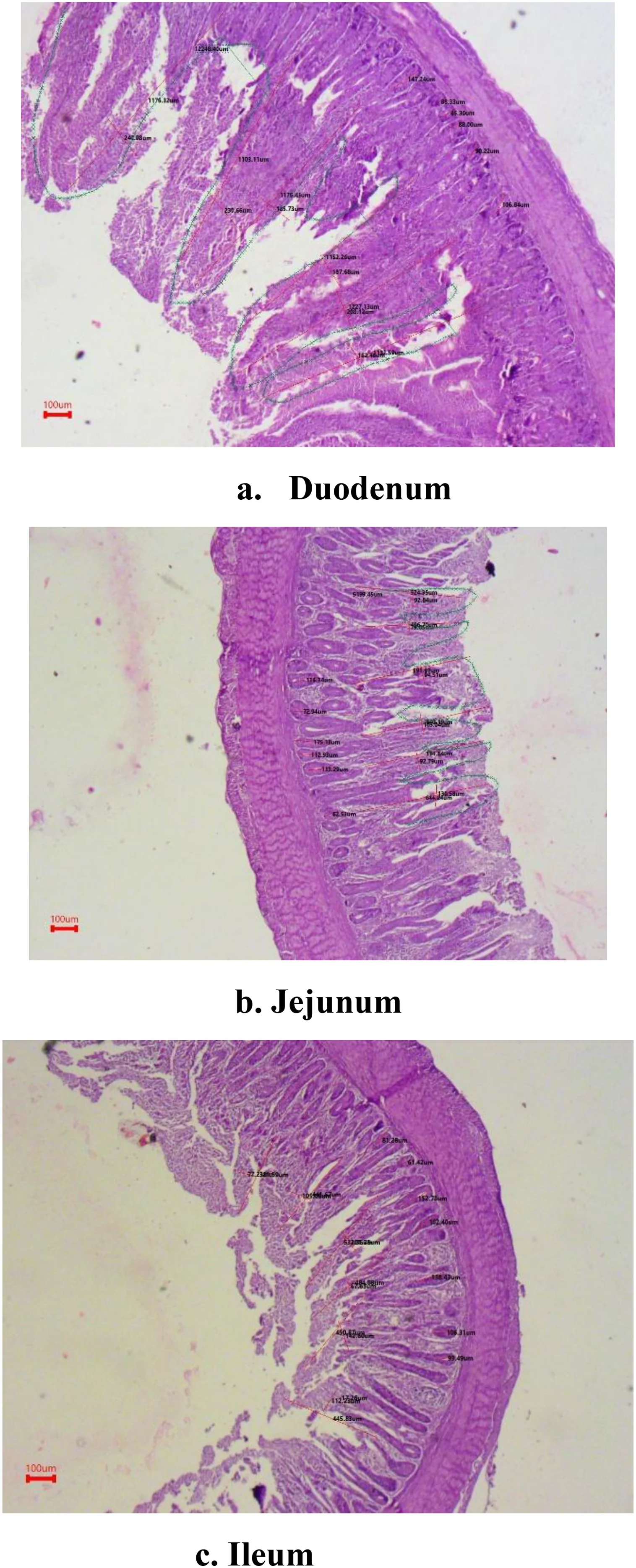
Histomorphometry of the small intestine [**(a)** duodenum; **(b)** jejunum; **(c)** ileum] of CARI-Debendra in the control group.

**Table 10:** Effect of different technological interventions during the embryonic and posthatch periods on intestinal biometry of CARI-Debendra chickens (*n* = 12).

Effects	Treatments	Sixth week							12th week						
		Intestine length (cm)	Intestine weight (%)	Duodenum (cm)	Jejunum (cm)	Ileum (cm)	Caecum-I (cm)	Caecum-II (cm)	Intestine length (cm)	Intestine weight (%)	Duodenum (cm)	Jejunum (cm)	Ileum (cm)	Caecum-I (cm)	Caecum-II (cm)
Main effect															
Prehatch manipulation	Control	120.75	5.45^a^	19.08	47.08	47.00	11.95	11.70	153.91	5.68	27.14	59.55	56.45	19.16	18.88
	ETC	119.94	4.42^b^	18.79	44.83	46.91	12.94	13.45	155.83	4.96	27.30	59.56	56.37	18.91	18.63
	*p*-value	0.89	0.02^*^	0.77	0.34	0.97	0.29	0.08	0.85	0.36	0.92	1.00	0.98	0.76	0.77
Posthatch manipulation	NPHHS	123.93	5.09	19.00	47.25	48.75	12.12	12.66	155.25	4.74	29.05	60.67	59.75	18.25	18.00
	EPHHS	121.56	4.52	19.31	46.50	48.68	13.03	12.90	163.61	6.52	27.00	62.37	58.50	20.31	20.00
	*p*-value	0.52	0.37	0.80	0.53	0.19	0.66	0.82	0.43	0.11	0.31	0.53	0.42	0.12	0.13
**Interaction effects**	Control	123.62	5.87	19.75	47.25	48.00	11.25	11.45	154.00	4.99	29.05	60.67	59.75	18.50	18.12
	Control + NPHHS	124.12	5.13	19.62	48.00	49.00	12.87	12.30	162.12	6.94	26.75	62.25	58.25	20.37	20.12
	ETC + NPHHS	124.25	4.31	18.25	47.25	49.50	13.00	13.87	156.50	4.50	29.05	60.68	59.75	18.00	17.87
	ETC + EPHHS	119.00	3.92	19.00	45.00	48.37	13.20	13.50	164.50	6.11	27.25	62.50	58.75	20.25	19.87
	*p*-value	0.87	0.46	0.53	0.78	0.91	0.81	0.87	0.99	0.97	0.99	0.99	0.99	0.97	1.00
	SEM	2.82	0.22	0.46	1.10	1.27	0.42	0.47	4.93	0.39	0.84	2.31	2.66	0.41	0.40

Means with the same superscript in a column do not differ significantly.

*ETC*, embryonic thermal conditioning; *NPHHS*, no posthatch herbal supplementation; *EPHHS*, early posthatch herbal supplementation; *n*, number of observations in each group.

^*^ *p* < 0.05—level of significance.

Interaction effect is the interaction between pre-hatch thermal conditioning and post-hatch performances.

**Figure 4:**
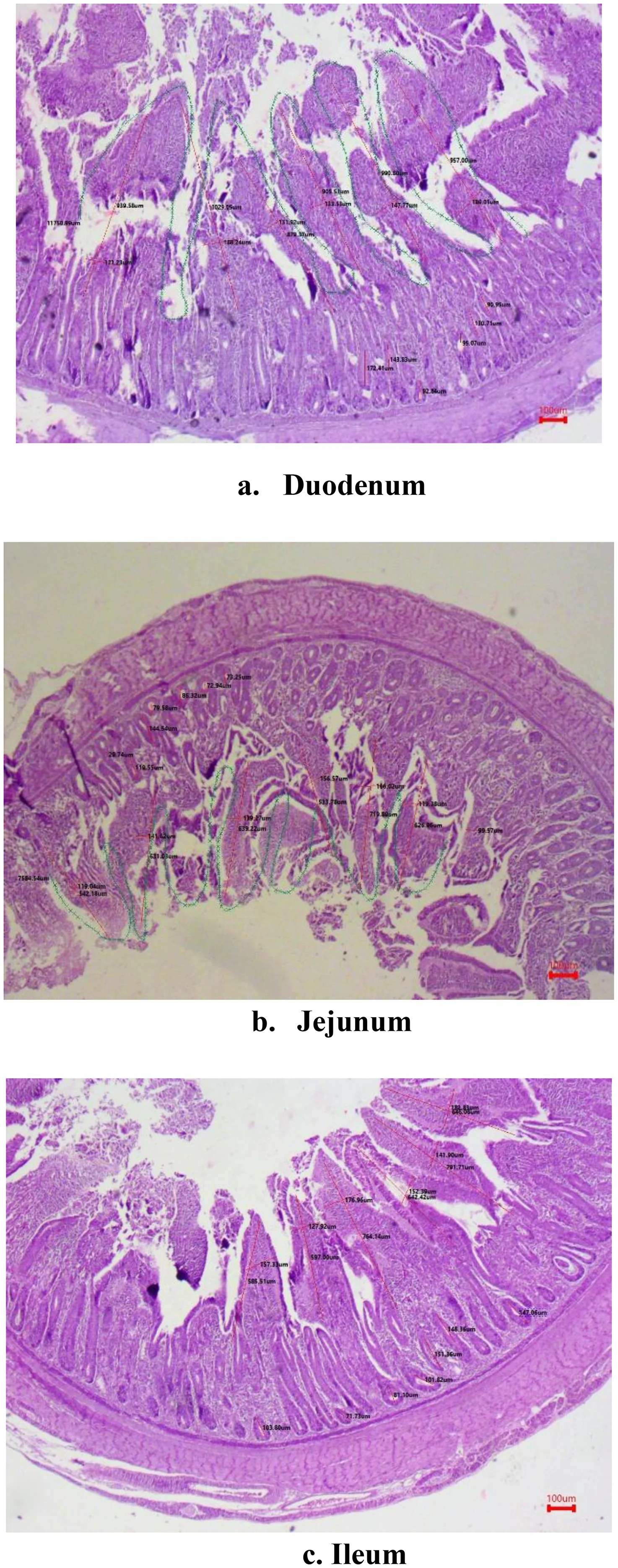
Histomorphometry of the small intestine [**(a)** duodenum; **(b)** jejunum; **(c)** ileum] in CARI-Debendra of the ETC group.

### 3.8 Biochemical parameters

Significant differences (*p* < 0.05) were observed in CK activity, T3 and T4 concentrations at 6 weeks between the control and thermally conditioned groups. Serum CK concentration was higher in the ETM group than that in the control, whereas there was no difference in LDH activity, indicating substantial variability in enzyme responses. In CARI-Debendra birds, thermal conditioning resulted in a significant decrease in T3 concentration and an increase in T4 concentration (*p* < 0.01) compared with the control (**Table 11**).

**Table 11:** Effect of thermal conditioning during the embryonic period on biochemical parameters of CARI-Debendra chicken (*n* = 8).

Parameters	Control	ETM
LDH (IU/L)	1,128 ± 417.30	788.03 ± 114.10
CK (IU/L)^*^	500^b^ ± 57.73	1,400^a^ ± 159.87
T3 (pg/dl)^**^	99.52^a^ ± 3.65	46.77^b^ ± 0.89
T4 (pg/dl)^**^	1.06^b^ ± 0.21	1.61^a^ ± 0.06
TSH (pg/dl)	0.004 ± 0.001	0.003 ± 0.001

Means with the same superscript in a column do not differ significantly.

*n*, number of observations in each group; *LDH*, lactate dehydrogenase; *CK*, creatine kinase; *T3*, triiodothyronine; *T4*. thyroxine, *TSH*, thyroid-stimulating hormone.

*p < 0.05; **p < 0.01—levels of significance.

## 4 Discussion

The UV-Vis and FTIR analyses of turmeric indicated the presence of curcumin, which has been reported to cause a reduction in oxidative stress (Saber et al., 2026), an improvement in antioxidant defences, and improvements in cecal inflammation and antioxidant capacity without altering cecal microbiota in broilers infected with *Eimeria tenella*, resulting in improved growth performance and anticoccidial index (Chen et al., 2024).

The UV-Vis analysis of ginger revealed the presence of gingerols and shogaols with antioxidant, anti-inflammatory, and antimicrobial properties (Mao et al., 2019), and FTIR analysis of ginger showed aromatic compounds such as zingiberene, which is responsible for the distinctive flavour and aroma and has antibacterial, antifungal, and antioxidant activities (Sharifi-Rad et al., 2017), and limonene, which modulates key inflammatory mediators and activates antioxidant pathways through Nuclear factor erythroid 2-related factor 2 (Nrf2) (Kathem et al., 2024).

The GC-MS chromatogram of *C. longa* (turmeric) showed the presence of acetic acid, 3,7,11,15-tetramethylhexadecyl ester, which was also found in the GC-MS analysis of *Adiantum capillus-veneris* L., a potential folklore therapeutic plant with antimicrobial activities (Kale, 2015). 1-Decanol, 2-hexyl-, present in *C. longa*, was also found in *Tagetes patula*, *Tagetes erecta*, and *Azadirachta indica* and has been reported to activate the Nrf2/HO-1 axis while simultaneously suppressing NF-κB activity, attenuating *Cutibacterium acnes*-induced inflammation (Shin et al., 2026). 1-Heptacosanol interacts with the immune-related proteins 1YN5 and 3RG1 through hydrophobic and electrostatic interactions and exhibits antibacterial activity (Thasmi et al., 2024).

*Zingiber officinale* (ginger) contains compounds identified by GC-MS, such as ethanone, 1-(2,2-dimethylcyclopentyl)-, which was also found in extracts of *Loriellopsis cavenicola* cyanobacteria from a freshwater pond in South Africa, exhibiting significant antioxidant activity (Ikhane et al., 2024). The compound 1-but-2-enylpyrrolidine contains significant features of the pyrrolidine ring and exhibits different biological profiles, making it a potential drug candidate (Li Petri et al., 2021). Also, compounds like *N*-butyric acid 2-ethylhexyl ester are butyric acid salts that can stimulate immunomodulatory activity by suppressing inflammation and restoring intestinal function, which may promote animal growth and development (Gerunova et al., 2024).

This study demonstrates that thermal conditioning during the embryonic stage can enhance the thermotolerance of slow-growing poultry varieties without negatively impacting hatchability or fertility. Similar observations have been reported in broilers by Yahav et al. (2004) and Loyau et al. (2013) under varying temperature regimes and exposure durations. Some studies report decreased hatchability due to elevated incubation temperatures, particularly during sensitive embryonic stages (Halle and Tzschentke, 2011). Collin et al. (2007) documented improved hatchability with exposure to 39.5 °C from embryonic days (E) 16 to 18 for 12 and 24 h. The absence of significant differences in hatchability in the present study may be attributed to the relatively mild increase in temperature and short exposure duration during mid-incubation in slow-growing birds.

Thermal conditioning appeared to accelerate the hatching process, with 80% of chicks hatching by 492 h compared with 502 h in the control group. Piestun et al. (2008) similarly reported earlier hatching in thermally treated embryos (12 h/day, E7–E16) compared with the control and continuously thermally manipulated groups. Lin et al. (2006) observed reduced hatch weight in ROSS-708 broilers exposed to 38.1 °C from E0 to E5, attributed to earlier hatch timing; however, female broilers in the heat-treated group had increased body weight at 14 days, suggesting no adverse dehydration effects. Other studies, including Piestun et al. (2013), have also indicated beneficial effects of thermal manipulation on hatch weight.

The synergistic effect of embryonic heat conditioning and posthatch herbal supplementation significantly enhanced thermotolerance and weight gain up to 6 weeks, underscoring the value of this dual approach. Lowered body temperature of thermally conditioned chicks and T3 concentration indicated a reduced metabolic rate and improved thermotolerance. Similar findings of lowered body temperature after thermal conditioning at 39.5 °C from E16 to 18 were observed in Ross broilers compared with controls by Collin et al. (2005). Herbal adaptogens such as turmeric and ginger have long been used in poultry for heat stress alleviation, feed intake stimulation, growth promotion, and immunomodulation (Elghalid et al., 2021). Turmeric is widely used in Ayurvedic medicine and exhibits potent antioxidant, anti-inflammatory, antimicrobial, and hepatoprotective properties. Thomas et al. (2020) reported improved body weight in Gramapriya birds supplemented with 0.5% turmeric and ginger. Similarly, El-Hack et al. (2020) demonstrated enhanced growth performance, immunity, gut morphology, egg quality, and economic efficiency with these supplements. Farinu et al. (2004) and Al-Sultan (2003) also reported improved growth in broilers with the inclusion of ginger and turmeric, respectively. However, Kafi et al. (2017) found better growth with 0.75% turmeric alone than with a 0.5% turmeric–ginger combination.

No significant difference in carcass characteristics, except thigh weight, was observed between the herbal-supplemented group and the control, similar to the findings of Wang et al. (2015). No significant differences in dressing weight, thigh weight, back, neck, and wing percentages were observed among the control and other treatment groups receiving 0%–0.75% turmeric and ginger combinations (Kafi et al., 2017). Exposure to high incubation temperature for E11–16 days stimulated liver growth, which undergoes the most rapid mitotic division before E12 of incubation (Dumm and Levy, 1949). El-Moniary et al. (2010) reported that early-age heat conditioning in the posthatch period, along with the feeding regimen, significantly improved liver weight compared with that in the nonheat-conditioned group. The inclusion levels of the herbal supplements may be increased by 1% each to obtain optimal effects on performance.

No significant effect of either embryonic or herbal supplements was observed on feed intake or FCR in CARI-Debendra birds. Feeding with 5–20g/kg of ginger did not influence the feed intake or FCR in layers, as reported by Zhao et al. (2011). Consistent with this, Akbarian et al. (2011) found that supplementation of ginger at 0.25%, 0.5%, and 0.75% in feed had no significant effect on FCR in layers. In contrast, Thomas et al. (2020) reported better FCR and improved feed intake with 0.5% supplementation of a ginger and turmeric combination compared with the control in a slow-growing layer variety. Ghasemi et al. (2021) demonstrated that supplementation of broiler diets with a phytonutrient mixture (PM) under different protein levels alleviated the negative effects of heat stress while also influencing growth performance and digestive enzyme activity. Wang et al. (2015) reported that the addition of turmeric rhizome powder at 100 and 200 mg/kg in the diet of slow-growing Wenchang chicken had no effect on FCR during 3–8 weeks but significantly (*p* < 0.05) improved FCR during 9–12 weeks.

There is limited literature on the effects of early posthatch thermal manipulation and herbal supplementation in slow-growing improved varieties of poultry, especially under tropical conditions. Hence, other studies on broilers or layers were considered. Similarly, Rajkumar et al. (2015) reported nonsignificant differences in FCR between naked neck and Punjab brown breeds exposed to high embryonic temperatures during the late incubation period. In contrast, Piestun et al. (2011) observed that intermittent thermal manipulation during development and maturation of the hypothalamus-pituitary (at E7–16 for 12 h) negatively affected FCR in broilers reared under hot posthatch conditions.

The herbal-supplemented group and control showed haematocrit values within the normal range, as well as heterophil and lymphocyte counts. Similarly, Kafi et al. (2017) reported no significant differences in haematology (Hb, PCV, ESR) among birds fed diets containing 0.5%–0.75% turmeric, ginger, and their combination, respectively. Generally, heterophils are particularly sensitive to stressors such as adrenocorticotropic hormone. Acute heat stress alters the proportion of leucocytes in the blood, leading to lower lymphocyte counts and higher heterophil counts, resulting in an increase in the H/L ratio (Altan et al., 2000; Mashaly et al., 2004). These findings corroborate that thermal conditioning and posthatch herbal supplementation effectively improved thermotolerance in slow-growing broilers, thereby not markedly affecting the differential counts.

No significant effects were found on immune organ weights or cell-mediated immune responses at 6 and 12 weeks, respectively. However, a reduction in spleen weight—a key peripheral immune organ—was observed in the embryonically conditioned group, consistent with Seto (1981). Although embryonic conditioning may limit immune development, early dietary herbal supplements have been reported to improve the immune response, weight of immune organs, and beneficial gut microbiota, as reported by Kadam et al. (2009). Polyherbal supplements in the early period have been reported to cause immunomodulation and reduced lipid peroxidation, especially under heat stress (Morovat et al., 2016).

Intestinal weights were lower in the thermal treatment group at 6 weeks. Christensen et al. (2001) similarly reported reduced intestinal weight (but not length) in turkey poults exposed to elevated incubation temperatures. Aksit et al. (2010) also observed no changes in intestinal length but reported altered weight and morphology due to heat stress. In the current study, there were no differences in the lengths of the duodenum, jejunum, ileum, or caeca at either 6 or 12 weeks. These findings align with reports by Aksit et al. (2010). Villus height and crypt depth measurements at 6 weeks showed no significant variation, reflecting consistent intestinal development. Uni et al. (1999) noted that VH and CD plateaued by 6 days posthatch in the duodenum and by 10 days posthatch in the jejunum and ileum. Lorenzi and Matos (2002) reported antimicrobial activity with 1% turmeric supplementation, which may help maintain gut microbial balance.

Significant differences were observed in CK activity and thyroid hormone concentrations (T3 and T4) at 6 weeks between the control and embryonic thermal manipulation (ETM) groups. In contrast, no significant differences in LDH activity, a reliable indicator of stress, were found between groups. This suggests minimal cellular damage, as reflected by the absence of intracellular enzyme leakage into the bloodstream, and indicates the development of acquired thermotolerance. However, serum CK concentration was higher in the ETM group compared with the control, reflecting variability in enzyme responses (Melesse et al., 2011). Dalab et al. (2022) reported increased expression of CK in embryonic thermal-conditioned chickens during the mid-incubation period (ED 7–11) in pectoral and thigh muscles without adversely affecting growth performance. Elevated serum CK levels were also reported in thermally challenged chickens at 35 days, as well as in groups thermally conditioned during incubation at ED7–11, ED 11–15, and ED 15–18, respectively. Al-Zghoul et al. (2015) similarly reported that thermal manipulation at 38 °C–40°C (65% RH) for 18 h daily from ED12 to ED18 reduced T3 levels without affecting T4 concentrations, CK, LDH activity, or electrolyte balance. Aksit et al. (2010) observed inconsistencies in T3 and T4 concentrations, including increases, decreases, or no changes at different time points. They further reported that heat increment at 39.5°C for 6 h daily from ED10 to ED18 elevated T4 but decreased T3 concentrations at hatch, while no differences were detected at 10- or 21-day posthatch, regardless of the brooding temperatures.

Thyroid hormones are central to regulating metabolic rate and thermoregulation, as demonstrated in thyroidectomized birds. In the present study, the reduction in T3 concentration likely contributed to lower metabolic heat production, thereby facilitating thermoregulation in CARI-Debendra chicks. Concurrently, the increase in T4 concentration in thermally treated birds suggests impaired conversion of T4 to T3, potentially due to downregulation of iodothyronine deiodinase type III (DIO3). These observations are consistent with the findings of Al-Zghoul et al. (2015).

## 5 Conclusion

This study improved the understanding of the interaction between embryonic thermal conditioning and herbal feed additives in a slow-growing poultry variety through a nutrienvironmental approach. The phytochemicals present in *C. longa* and *Z. officinale* exhibit antioxidant, anti-inflammatory, antimicrobial, and immunomodulatory properties that can be helpful for weight gain, heat stress alleviation, feed intake stimulation, and immunomodulation in poultry through enhanced growth performance, egg quality, and economic efficiency. The findings highlight the potential of thermal conditioning, in combination with herbal supplementation, to improve body weight, enhance gut health, and modulate certain biochemical parameters (T3, T4, and TSH) indicative of thermotolerance in poultry, particularly in the context of climate change. The present study provides an opportunity to reevaluate natural feed additives and develop markers of thermotolerance, which may help mitigate the evolving challenges of heat stress in tropical regions.

## Data Availability

The original contributions presented in the study are included in the article/supplementary material. Further inquiries can be directed to the corresponding authors.
